# 

**Published:** 1905-04-15

**Authors:** 


**IMPORTANT NOTICE.**

**On account of the Easter Holidays, we beg to announce that we shall go to press with our issue of the 22nd APRIL on TUESDAY NIGHT, the 18th inst. Advertisements intended for insertion in this issue should therefore reach us by 4 p.m. ON TUESDAY, the 18th inst.**

**NOTICE TO CORRESPONDENTS.**

All MSS., Letters, Books for Review, and other matters intended for the Editor should be addressed THE EDITOR, "THE HOSPITAL," THE HOSPITAL BUILDINGS, 28 & 29 SOUTHAMPTON STREET, STRAND, W.C.

The Editor cannot undertake to return rejected MS., even when accompanied by stamped directed envelope.

**Notice to Advertisers and Subscribers.**

All Advertisements, Orders for Copies of THE HOSPITAL, and Business Communications should be addressed to **THE MANAGER (Not to the Editor),** THE HOSPITAL, 28 & 29 Southampton Street, Strand, London, W.C.

The Cost of Subscription, post free, is at follows:—Per week, 3½d.; per quarter, 3s. 3d.; per half-year, 6s. 6d.; per annum, 13s. Foreign and Colonial:—Per annum, 19s. 6d., payable, in all cases, in advance.

**NOTICE.—Vol. XXXVII. of "The Hospital" October 1904 to March 1905), in handsome cloth binding, will shortly be on sale at the office of this Journal, price 10s. 6d. Cases for binding the Numbers contained in this Volume 2s. Index to Vol. XXXVII., 3½d., post free.**

**The Monthly Part for April is now ready. Price 1s., by post, 1s. 3d.**

### BOOKS RECEIVED.

T. Fisher Unwin.

"Studies in General Physiology." Two Vols. By Jacques Loeb.

Rebman, Limited.

"Tropical Light." By Major Chas. E. Woodruff, M.D.

Baillière, Tindall and Cox.

"The Conjunctiva in Health and Disease." By A. Bishop Harman, M.B.Cantab., F.R.C.S.Eng.

"Clinical Lectures on Appendicitis, Radical Cure of Inguinal Hernia and Perforating Gastric Ulcer." By G. R. Turner, F.R.C.S.

"Surface Anatomy." By T. G. Moorhead, M.D.

T. Nelson and Sons.

"Harmsworth Encyclopædia." Parts 1, 2, and 3. (In advance.)

Spottiswoode and Co.

"The Medical Register, 1905."

Elliot Stock.

"Decorating and Furnishing." By T. Myddelton Shallcross.

G. W. Bacon and Co., Limited.

"Cold Catching, Cause, and Cure." By G. W. Bacon, F.R.G.S

C. Griffin and Co., Limited.

"Foods and Dietaries." By R. W. Burnet, M.D.

### Medical Appointments.

**C. A. Anderson**, B.A.Cantab., Senior House Physician to St. Bartholomew's Hospital. **H. J. D. Birkett**, B.A.Cantab., Senior House Surgeon to St. Bartholomew's Hospital. **E. A. R. Bligh**, M.B.Syd., Resident Medical Officer at the Sydney Hospital, Australia. **R. H. Bott**, M.R.C.S., L.R.C.P.Lond., Junior House Surgeon to St. Bartholomew's Hospital. **A. W. Brodribb**, M.A., B.M., B.Ch.Oxon., Junior House Physician to St. Bartholomew's Hospital. **J. Burfield**, M.B., B.S.Lond., Senior House Surgeon to St. Bartholomew's Hospital. **G. H. Colt**, B.A., M.B., B.C. Cantab., Senior House Surgeon to St. Bartholomew's Hospital. **A. W. D. Coventon**, M.A., B.C.Cantab., Junior House Surgeon to St. Bartholomew's Hospital. **W. L. Cripps**, B.A., B.C.Cantab., Senior House Surgeon to St. Bartholomew's Hospital. **W. J. Cumberlidge**, B.A.Cantab., Junior House Surgeon to St. Bartholomew's Hospital. **R. B. Etherington-Smith**, M.B., B.C. Cantab., Extern Midwifery Assistant at St. Bartholomew's Hospital. **C. H. Fielding**, M.R.C.S., L.R.C.P.Lond., Junior House Physician to St. Bartholomew's Hospital. **A. E. Finckh**, M.B. Syd., Resident Medical Officer at the Sydney Hospital, Australia. **H. P. Gibb**, B.A.Cantab., Junior House Surgeon to St. Bartholomew's Hospital. **Professor A. S. Grünbaum**, M.D.Cantab., F.R.C.P.I., Honorary Pathologist to the Royal Infirmary, Leeds. **C. F. Hadfield**, M.A.Cantab., Senior House Physician to St. Bartholomew's Hospital. **F. A. Hepworth**, M.A., M.B., B.C. Cantab., Senior House Physician to St. Bartholomew's Hospital. **T. G. Hine**, M.A., B.C.Cantab., Junior House Physician to St. Bartholomew's Hospital. **Gordon M. Holmes**, M.D.Dub., Director of Neuro-pathological Research, National Hospital for the Paralysed and Epileptic. **B. Hudson**, M.A., M.B., B.C. Cantab., Senior House Physician to St. Bartholomew's Hospital.

### BOOKS.

There are hundreds of books on NURSING and the various branches of this important Profession. Some are good, but many are indifferent and practically useless from an Educational standpoint. It is our aim to help NURSES to discriminate between the wheat and the tares, and to supply only the latest and best works on NURSING and such Subjects as are allied to Nursing. We can tell you the latest book on any subject, or anything about a particular book whether published by ourselves or others, and shall be pleased to supply the information if you will write to

THE SCIENTIFIC PRESS LTD.,
28 & 29 Southampton Street, Strand,
LONDON, saying what you require. No request is too insignificant to receive our careful and prompt attention, and no charge is made for the information supplied.

SEND FOR OUR LATEST CATALOGUE ON NURSING MANUALS.

**THE SCIENTIFIC PRESS LTD.**

**28/29 Southampton St.,
Strand, LONDON.
W.C.**

